# Progranulin and neuropathological features of Alzheimer’s disease: longitudinal study

**DOI:** 10.1007/s40520-024-02715-9

**Published:** 2024-03-05

**Authors:** Fardin Nabizadeh, Rasa Zafari, Alzheimer’s disease Neuroimaging Initiative (ADNI)

**Affiliations:** 1 School of Medicine, Iran University of Medical Sciences, Tehran, Iran; 2 Department of Neurology, Iran University of Medical Sciences, Tehran, Iran; 3 School of Medicine, Tehran University of Medical Science, Tehran, Iran

**Keywords:** Alzheimer’s disease, Amyloid βeta, Tau, Progranulin, Microglia

## Abstract

**Background:**

Progranulin is an anti-inflammatory protein that plays an essential role in the synapse function and the maintenance of neurons in the central nervous system (CNS). It has been shown that the CSF level of progranulin increases in Alzheimer’s disease (AD) patients and is associated with the deposition of amyloid-beta (Aβ) and tau in the brain tissue. In this study, we aimed to assess the longitudinal changes in cerebrospinal fluid (CSF) progranulin levels during different pathophysiological stages of AD and investigate associated AD pathologic features.

**Methods:**

We obtained the CSF and neuroimaging data of 1001 subjects from the ADNI database. The participants were classified into four groups based on the A/T/N framework: A + /TN + , A + /TN−, A−/TN + , and A−/TN−.

**Results:**

Based on our analysis there was a significant difference in CSF progranulin (*P* = 0.001) between ATN groups. Further ANOVA analysis revealed that there was no significant difference in the rate of change of CSF-progranulin ATN groups. We found that the rate of change of CSF progranulin was associated with baseline Aβ-PET only in the A−/TN + group. A significant association was found between the rate of change of CSF progranulin and the Aβ-PET rate of change only in A−/TN +

**Conclusion:**

Our findings revealed that an increase in CSF progranulin over time is associated with faster formation of Aβ plaques in patients with only tau pathology based on the A/T/N classification (suspected non-Alzheimer’s pathology). Together, our findings showed that the role of progranulin-related microglial activity on AD pathology can be stage-dependent, complicated, and more prominent in non-AD pathologic changes. Thus, there is a need for further studies to consider progranulin-based therapies for AD treatment.

**Supplementary Information:**

The online version contains supplementary material available at 10.1007/s40520-024-02715-9.

## Introduction

Alzheimer’s disease (AD) is considered the most common cause of dementia among the older population with a prevalence of 30% in patients over 60 years old [1, 2]. AD starts with episodic memory loss followed by progressive cognitive decline which in turn can cause disabilities in daily activities [3]. AD is characterized by neuronal loss, dysfunction of synapses, neuroinflammation, and the pathologic deposition of beta-amyloid (Aβ) plaques and phosphorylated tau (P-tau) fibrils [4]. The Aβ peptide is involved in the hemostasis process of the brain and originates from the amyloid precursor protein (APP) gene [5, 6]. It is believed that tau along with Aβ plays an important role in the pathology of AD. Tau is involved in the maintenance of synaptic functions which in turn plays an important role in cognitive functions [7, 8].

The accumulation of amyloid plaques and tau proteins can activate the microglia and as a result, they aggregate around the amyloid plaques which in turn exacerbates disease severity [9, 10]. In response to neuroinflammation, as happens in AD, microglia express particular proteins such as progranulin [11]. Progranulin is an anti-inflammatory protein that originates from the GRN gene. This protein plays an essential role in the synapse function and the maintenance of neurons in the central nervous system (CNS) [12, 13]. The expression of this protein by microglia increases in pathologic conditions [14]. It has been shown that the CSF level of progranulin increases in AD patients [15]. Moreover, the accumulation of progranulin in the margins of amyloid plaques is reported in mouse model studies [16]. Recent studies reported that there is a significant association between amounts of progranulin and the accumulation of Aβ and tau in the brain tissue [17, 18]. Some of these studies reflected that the overexpression of progranulin decreases the risk of Aβ and tau depositions which can decelerate the process of cognitive decline [17, 19].

To better understand the role of mentioned biomarkers in the AD pathophysiology the Amyloid/Tau/Neurodegeneration (A/T/N) system was provided by the National Institute on Aging and the Alzheimer’s Association [8]. In this classification system, A refers to amyloid plaques, T refers to tau neurofibrillary tangles which are two the most reliable indicators of AD, and N in the A/T/N system represents neurodegeneration. These factors can be evaluated through a variety of imaging and enzyme immunoassays (EIAs) methods [20–22]. Previous studies reflected contradictory findings in the association of progranulin and cerebrospinal fluid (CSF) Aβ and tau and they lacked longitudinal assessment on the role of progranulin in AD pathology. In this study, we aimed to investigate the association of progranulin and longitudinal change in the AD imaging biomarkers including Aβ- and tau-PET to provide a better view on the role of progranulin in the AD pathophysiology.

## Methods and materials

### Participants

We performed a cross-sectional and longitudinal study that investigates the levels of CSF progranulin in a cohort consisting of 1001 participants with available baseline CSF-progranulin measures enrolled in the Alzheimer’s Disease Neuroimaging Initiative (ADNI) (http://adni.loni.usc.edu). The ADNI project, led by Principal Investigator Michael W Weiner, is a multicenter longitudinal study aimed at the development and validation of biomarkers for subject selection and as surrogate outcome measures in late-onset AD. The study procedures were approved by the institutional review boards (IRB) of all participating centers, and informed consent was obtained from all participants or their surrogates. In addition, the study was approved by our local IRB (LMU).

### Classification

In accordance with the recently published 2018 NIA-AA “research framework” for the diagnosis of AD [20], participants enrolled in the ADNI project were categorized into distinct groups based on their biomarker profiles, utilizing the A/T/N scheme [23]. The A/T/N scheme encompasses three biomarker groups: “A” represents aggregated Aβ, “T” represents aggregated tau, and “N” represents neurodegeneration. Each biomarker group was classified as either negative (−) or positive ( +) based on the normal or abnormal status of the respective biomarkers.

In this study, participants were classified as “A + ” if their CSF Aβ1-42 level was below 976.6 pg/ml, “T + ” if their P-tau181 level was higher than 21.8 pg/ml, and “N + /” if their T-tau level surpassed 245 pg/ml. For the purpose of simplifying comparisons, we combined the aggregated tau (T) and neurodegeneration (N) groups. Consequently, TN negative (TN−) was defined as having both aggregated tau (T) and neurodegeneration (N) biomarkers falling within the normal range (T− and N−, specifically P-tau181P ≤ 21.8 pg/ml and T-tau ≤ 245 pg/ml). Participants were classified as TN positive (TN +) if either the aggregated tau (T) or neurodegeneration (N) biomarkers were abnormal (T + or N + , i.e., P-tau181P > 21.8 pg/ml or T-tau > 245 pg/ml). It is noteworthy that only a small proportion, specifically 5.4%, of the total individuals exhibited discrepancies between the aggregated tau (T) and neurodegeneration (N) biomarker groups.

### CSF-progranulin measurements

CSF-progranulin measurements were performed using MSD platform-based assay, which has been previously documented [24, 25]. CSF-progranulin levels were measured using an established ELISA protocol. Streptavidin-coated plates were blocked overnight, then incubated with a biotinylated anti- progranulin capture antibody, followed by washing. CSF and internal standard samples were added and incubated, then washed. A detection antibody was added, followed by washing and incubation with a secondary antibody. After washing, an electrochemical signal was generated using MSD Read buffer, and light emission was measured. Recombinant human progranulin protein served as a standard. progranulin levels were reported as pg/mL. The CSF-progranulin measurements used in this study are accessible to the public through the ADNI database. Number of participants with available CSF progranulin in each time-point is detailed in Supplementary 1.

### PET imaging

Within the ADNI study, AV45-PET (Aβ) imaging was conducted using a total of 6 5-min time frames, approximately 60–90 min after the injection of 370 Mbq radiolabeled F18-AV45 tracer. These time frames were subsequently co-registered and averaged to generate a mean AV45 image. The global AV45 standardized uptake value ratio (SUVR) was calculated by averaging across specific cortical regions and normalizing to a composite reference region that encompassed the entire cerebellum and cerebral white matter. This methodology adheres to a previously described protocol, which demonstrated the stability of longitudinal AV45 changes using this composite reference region [26]. To evaluate longitudinal changes in AV45 uptake, we computed the annual rate of change in global AV45 SUVR values for each participant. This involved determining the absolute difference in SUVR between subsequent assessments and dividing it by the time difference in years. For participants with more than two AV45 scans, multiple AV45 change rates were calculated, such as from baseline to any available follow-up visit.

Tau-PET imaging was performed 75 min after the administration of F18-radiolabeled AV1451 tracer, utilizing 6 5-min blocks. The acquired images were co-registered and averaged across blocks, and then intensity-normalized to the inferior cerebellar gray matter, following the methodology outlined in Maass et al. [27]. Specific SUVR scores for regions of interest (ROIs) defined by Braak stages were obtained from the ADNI core and downloaded from the ADNI database. Detailed protocols for these procedures can be found on the ADNI homepage and in previous publications [28]. It is important to note that we excluded Braak stage 2 (i.e., hippocampus) from the analysis due to known off-target binding of the AV1451 tracer in this particular region, as reported in Lemoine et al. [29]. For participants with more than two AV1451 scans, multiple AV1451 change rates were calculated, such as from baseline to any available follow-up visit.

### Cognitive assessment

Memory performance was assessed using the ADNI-mem composite memory score, which encompasses various cognitive tests. These tests include the Rey Auditory Verbal Learning Test, AD Assessment Scale—Cognitive Subscale, Word Recall of the Mini-Mental State Examination (MMSE), and the Wechsler Logical Memory Scale II. The ADNI-mem score combines the results of these tests to provide a comprehensive evaluation of memory function [30].

### Statistical analysis

All statistical analyses were conducted using the R software (https://www.rproject.org/). The normality of the data was assessed using the Kolmogorov–Smirnov test. Variables that did not follow a normal distribution were log(10) transformed prior to further analysis. Baseline sociodemographic data were compared between the ATN groups using analysis of variance (ANOVA), while Chi-square tests were employed for comparing dichotomous variables such as APOE status and sex. To account for multiple comparisons, Bonferroni correction was applied to all post-hoc tests.

To evaluate the rate of change over time in CSF-progranulin concentrations, individual slopes were derived for each subject using a linear mixed effect model (LMEM) from the lme4 R package. The LMEM included time (years from baseline) as a fixed factor and subject as a random factor. Subsequently, ANOVA was utilized to compare biomarker slopes and baseline levels between the ATN groups. Linear regression models were employed to investigate whether baseline concentration and the rate of change in CSF-progranulin level predicted ADNI-mem and PET tau and Aβ load. All analyses were adjusted for the effects of age, sex, education, and APOE ε4. Linear regression analyses were performed to assess the association between CSF progranulin (baseline and slope) and tau and Aβ-PET (baseline and rate of change) for the entire cohort and within individual-ATN groups. To control for false positives, the Bonferroni correction method was applied, with a significance level set at *P* < 0.05.

## Results

The demographic and clinical characteristics of the included subjects are detailed in Table 1.

**Table1.** Demographic and clinical characteristics

Variable	A-/TN- (*n* = 246)	A + /TN- (*n* = 166)	A + /TN + (*n* = 407)	A-/TN + (*n* = 182)	*P*-value
Age (years)	71.2 ± 6.8	73.0 ± 7.0	73.7 ± 7.4	74.0 ± 7.6	< 0.001
Female (%)	113 (46%)	53 (32%)	184 (45%)	88 (48%)	0.008
Education (years)	16.2 ± 2.7	16.2 ± 2.8	15.8 ± 2.8	16.0 ± 2.6	0.201
APOEε4 carriers (%)	43 (17%)	77 (36%)	300 (73%)	50 (27%)	< 0.001
MMSE	28.6 ± 1.5	27.6 ± 2.3	25.9 ± 2.8	28.1 ± 2.1	< 0.001
CSF progranulin (pg/ml)	1461.6 ± 579.4	1302.5 ± 380.5	1604.6 ± 347.0	1429.9 ± 374.2	< 0.001
MCI (%)	147 (60%)	101 (61%)	225 (55%)	111 (61%)	0.112

Data are presented as mean ± standard deviation unless specified otherwise

*APOEε4* apolipoprotein E genotype (carrying at least one ε4 allele), *CSF* cerebrospinal fluid, *MMSE* Mini-Mental State Evaluation, *MCI* mild cognitive impairment, *A* Aβ pathology, *TN* Tau neurodegeneration

### Baseline differences of CSF-progranulin concentrations between the ATN groups

**Figure1:**
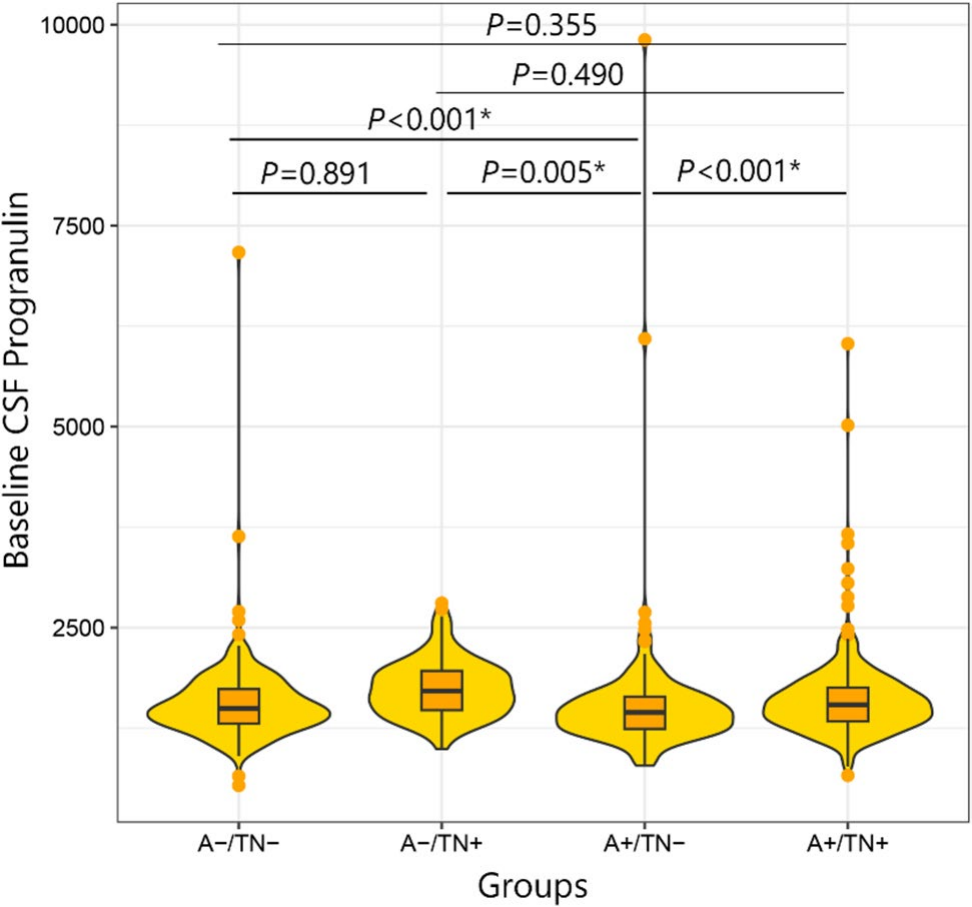
Baseline level of CSF progranulin

Based on our analysis there was a significant difference in CSF progranulin (*P* = 0.001) between ATN groups (Fig. 1). The post-hoc pairwise comparisons showed a significant difference between groups for CSF progranulin (*P* < 0.001)(A−/TN− vs A + /TN−, A + /TN− vs A−/TN + , and A + /TN− vs A + /TN +).

### Rate of change of CSF-progranulin concentrations between the ATN groups

**Fig. 2:**
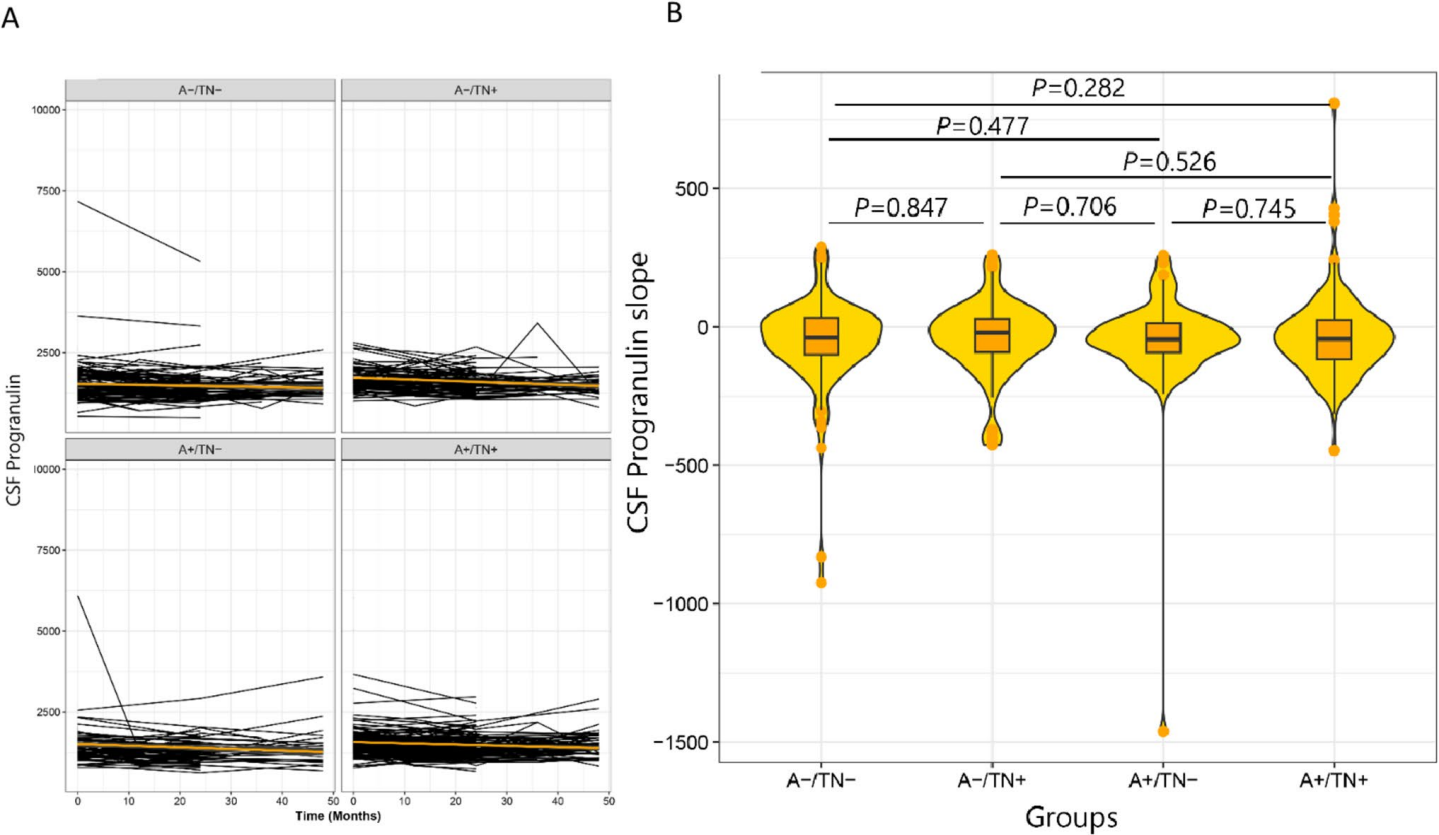
**A** longitudinal trajectories of CSF progranulin and **B** rate of change of CSF progranulin

We estimated the trajectory of CSF progranulin over 4 years of follow-up across the ATN group using linear mixed-effect models adjusted for the effect of age, sex, and APOE ε4 (Fig. 2A). Further ANOVA analysis revealed that there was no significant difference in the rate of change of CSF-progranulin ATN groups. The Bonferroni post-hoc pairwise comparisons showed the same results (Fig. 2B).

### Association between cognitive performance and CSF progranulin

**Fig. 3:**
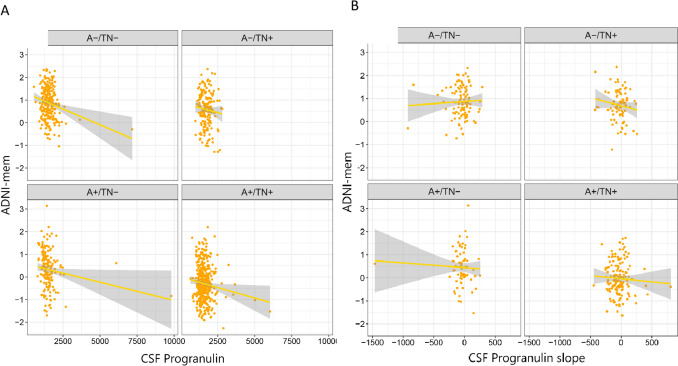
**A** association between baseline level of CSF progranulin and **B** rate of change of CSF progranulin with cognitive performance (ADNI-mem)

Using linear regression models adjusted for the effect of age, sex, education, and APOE e4 we found that the baseline level of CSF progranulin was significantly associated with lower cognitive performance in A-/TN- (*β* = − 0.152, *P* = 0.013) and A + /TN + (*β* = − 0.102, *P* = 0.041) (Fig. 3A). Among all participants, there was a significant association between CSF progranulin and cognitive performance (*β* = − 0.104, *P* < 0.001).

We analyzed the association between cognitive performance (ADNI-mem) and rate of change of CSF-progranulin concentrations using linear regression adjusted for the effect of age, sex, education, and APOE ε4. There was no significant association between cognitive performance (ADNI-mem) and the rate of change of CSF-progranulin concentrations in ATN groups (Fig. 3B).

### Association between Tau- and Aβ-PET with CSF progranulin

**Fig. 4:**
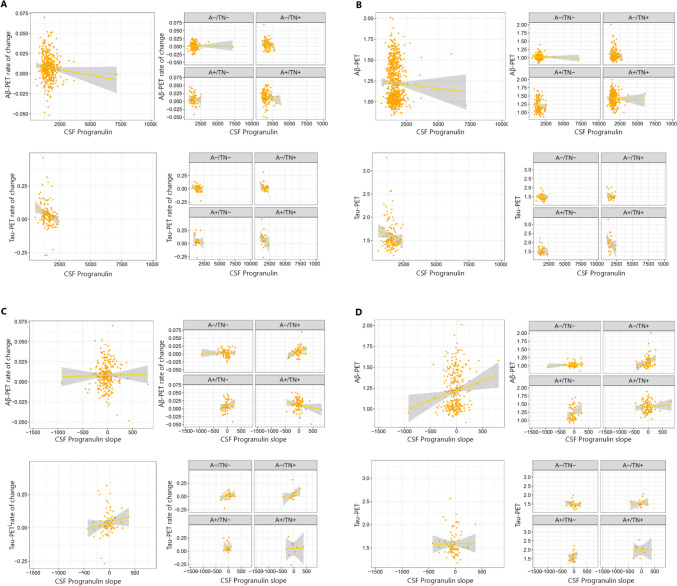
Association between baseline CSF progranulin and tau and Aβ-PET rate of change (**A**) and baseline tau and Aβ-PET (**B**). association between rate of change of CSF progranulin and tau and Aβ-PET rate of change (**C**) and baseline tau and Aβ-PET (**D**)

In the first step, we investigated the association between baseline neuroimaging findings including tau and Aβ-PET with baseline levels of CSF progranulin (Fig. 4B). There was no association between CSF progranulin and tau and Aβ-PET in ATN groups or the entire cohort (Table 2). Next, we aimed to assess the association between longitudinal changes in tau and Aβ-PET (rate of change) with baseline levels of CSF progranulin. Our analysis revealed that there was no association between CSF progranulin and Aβ-PET rate of change in ATN groups or entire cohorts (Fig. 4A). However, CSF progranulin was associated with the tau-PET rate of change in the entire cohort but not ATN groups.

**Table 2:** Association of ADNI-mem, tau (flortaucipir) and Aβ (florbetapir) uptake for CSF progranulin baseline concentrations and rates of change over time (slope)

Variable	Entire cohort (*n* = 1001)		A-/TN- (*n* = 246)		A + /TN- (*n* = 166)		A-/TN + (*n* = 182)		A + /TN + (*n* = 407)	
	β	*P*-value	β	*P*-value	β	*P*-value	β	*P*-value	β	*P*-value
ADNI-mem										
CSF progranulin	− **0.104**	** < 0.001**	− 0.152	0.013	− 0.141	0.06	0.006	0.933	− 0.103	0.041
CSF-progranulin slope	− 0.011	0.823	0.028	0.775	− 0.024	0.847	− 0.14	0.194	− 0.023	0.778
Aβ-PET										
CSF progranulin	− 0.015	0.656	− 0.004	0.959	− 0.046	0.618	− 0.159	0.059	− 0.027	0.674
CSF-progranulin slope	0.092	0.089	0.08	0.514	0.022	0.867	**0.306**	**0.015**	0.109	0.317
Tau-PET										
CSF progranulin	− 0.02	0.351	− 0.109	0.465	− 0.038	0.827	− 0.167	0.315	− 0.245	0.231
CSF-progranulin slope	− 0.017	0.864	− 0.17	0.364	0.128	0.541	− 0.03	0.904	0.443	0.321
Aβ-PET rate of change										
CSF progranulin	− 0.054	0.26	0.034	0.706	− 0.196	0.118	− 0.205	0.053	− 0.097	0.298
CSF-progranulin slope	− 0.022	0.728	− 0.005	0.97	0.048	0.767	**0.311**	**0.023**	− 0.186	0.09
Tau-PET rate of change										
CSF progranulin	**− 0.207**	**0.016**	− 0.262	0.092	− 0.052	0.8	− 0.264	0.145	− 0.269	0.246
CSF-progranulin slope	0.138	0.238	0.29	0.136	− 0.045	0.855	0.146	0.213	− 0.248	0.312

Analyses adjusted for age, sex and APOE e4/PET scan. Baseline cognition was included in the model when predicting ADNI-mem at follow-up

*Aβ* amyloid β, A + /− Amyloid-β positive/negative, *ADNI* Alzheimer’s Disease Neuroimaging Initiative, *ATN* Aβ deposition, tau pathology and neurodegeneration, *β* beta, *β* standardized beta, TN + /− Tau/Neurodegeneration positive/negative

We analyzed if the longitudinal change in CSF progranulin (slope) was associated with baseline tau and Aβ-PET(Fig. 4D). We found that the rate of change of CSF progranulin was associated with baseline Aβ-PET only in the A−/TN + group. There was no significant association between longitudinal change in CSF progranulin with baseline tau-PET. We then investigated the possible association between the rate of change of CSF progranulin with longitudinal changes in tau and Aβ-PET (rate of change). A significant association was found between the rate of change of CSF progranulin and the Aβ-PET rate of change only in A−/TN + (Fig. 4C).

## Discussion

In this study, we evaluated the potential association between CSF progranulin and the level of Aβ and tau deposition in the AD continuum. Considering the increased expression of progranulin by microglia in patients with AD continuum, the results of our study reported significant alterations of CSF progranulin between different ATN groups. Moreover, linear regression provided a considerable association between progranulin level and cognitive performance in all participants. In terms of the rate of progranulin change, no apparent differences were observed between ATN groups. Also, it is shown that cognitive performance is not related significantly to the rate of CSF-progranulin change in ATN groups. Furthermore, there was no significant association between CSF progranulin and the rate of change in Aβ-PET. However, in patients with only tau pathology, we observed a significant association between the rate of change of CSF progranulin and the Aβ-PET rate of change.

Our study suggested that the level of CSF progranulin is significantly distinct between different ATN groups. However, Suárez-Calvet et al. in their study, provided that progranulin level alone cannot be a valuable predictor in AD diagnosis [15]. These findings are consistent with the results of another study assessing CSF-progranulin levels in frontotemporal dementia (FTD) [31]. There are other molecules involved in AD pathology as well as progranulin, some of which are associated with progranulin CSF level. It is shown that higher amounts of CSF progranulin are related to higher levels of triggering receptor expressed on myeloid cells 2 (TREM2) in CSF and associated with weaker cognitive performance in patients with late AD. Thus, they concluded that measuring both CSF progranulin and TREM2 can be a more reliable indicator of microglia activity in AD patients [15]. Moreover, studies trying to find a therapy for FTD by modifying the levels of progranulin were unsuccessful [32]. TREM2 is expressed by microglia of CNS and is involved in phagocytosis and the migration of microglia [33–35]. The roles of progranulin and TREM2 are against each other since the loss of TREM2 leads to the maintenance of microglia in a hemostatic stage. In contrast, the deficiency in progranulin increases the activity of microglia [34]. Chen et al., in their study, reported a significant association between TREM2 and cognitive decline in AD pathology-positive participants. However, this study reflected no reliable value for CSF progranulin in the diagnosis or the prognosis of AD [36]. Previously we found that the CSF soluble TREM2 is associated with a decrease in tau aggregate spreading through functional connection [37].

Morenas-Rodríguez et al. in their study evaluating the role of CSF progranulin in neurodegenerative diseases, reported no significant association between this biomarker and the performance of patients with different neurodegenerative disorders in MMSE assessment nor cortical atrophy algorithm in these patients [31]. None of these studies attempted to evaluate the association of CSF progranulin and AD longitudinally and this can be a reason for different results with our study. Animal studies suggested that CSF-progranulin insufficiency is correlated to the increased risk of Aβ deposition and the phosphorylation of tau protein in mouse models and it is involved in the progression of AD pathology in these animals [17, 18]. Another longitudinal study observing potential associations between imaging alterations and progranulin level in FTD patients revealed that patients with progranulin mutations reflect a faster rate of whole-brain atrophy and there is a significant correlation between the progranulin level and asymmetric atrophies in the inferior frontal, temporal, and inferior parietal lobe of gray matter [38]. The findings of Whitwell et al. supported the theory of rapid brain atrophy in patients with GRN mutations. This study also revealed that these patients reflect hippocampus atrophy at the same rate as patients with microtubule-associated protein tau (MAPT) gene mutations [39]. Some studies reported that brain atrophy and cortical thickness can be more reliable indicators of cognitive decline than other biomarkers such as progranulin or tau [36, 40]. Also, studies evaluating the association of progranulin and tau deposition in cognitive decline have shown that reduced levels of CSF progranulin in AD are associated with increased amounts of T-tau in these patients suggesting that progranulin prevents tau accumulation and in turn the risk of neurodegeneration [41]. Suárez-Calvet et al. reported a significant association between CSF progranulin and CSF T-tau and P-tau in patients with Alzheimer’s continuum category [15]. Our study revealed no significant associations between longitudinal changes in CSF progranulin and baseline tau-PET.

Our study also revealed that increased longitudinal changes of CSF progranulin are associated with increased Aβ-PET alterations over time only in A−/TN + . Patients with either increased tau or neurogenerative diseases and no evidence of Aβ deposition are considered A−/TN + who are recently defined as suspected non-Alzheimer’s pathology (SNAP) and reflect a non-AD related neurodegeneration [42, 43]. This phenotype is more common in older males and reflects lower amounts of APOE 4 [42, 44, 45]. It is shown that progranulin decreases the accumulation risk of Aβ and thus, loss of this protein is associated with increased Aβ plaques. A significant association was reported between CSF progranulin and CSF Aβ in AD patients [46]. Revuelta et al. in their immunohistochemistry (IHC) study also reported a positive correlation between beta-amyloid concentration and progranulin [47].

Previous studies reported that increased activity of microglia is related to the progression of neurodegenerative disorders [48]. Progranulin is one of the main proteins expressed by microglia that is involved in the function of lysosomes in these cells and the reduction of progranulin levels results in increased expression of genes controlling the function of lysosomes [49]. Moreover, it is suggested that progranulin can play an essential role in the formation of lysosomes in microglia [12]. In fact, this protein can significantly decrease the rate of brain aging by suppressing microglial activity through increasing lysosomal trafficking in these cells [50]. Results of our study showed no strong associations between CSF level of progranulin and the pathophysiology process of AD continuum which in turn denies the effect of progranulin-related microglial activity in early AD.

Considering the crucial role of progranulin in lysosomal function, neuronal viability, and CNS inflammation, the restoration of progranulin levels to their physiological range in individuals with granulin mutations holds promising potential as a therapeutic approach [51]. By addressing the deficiency of progranulin, this strategy aims to mitigate the detrimental consequences associated with impaired lysosomal function, neuronal loss, and inflammatory processes within the CNS. Thus, the normalization of progranulin levels may offer significant therapeutic benefits for patients affected by granulin mutations. However, despite the findings of other studies regarding the elevation or restoring the level of progranulin, our findings does not support the role of progranulin in the AD pathophysiology progression [15, 52–54]. Thus, there might be need for further studies with larger sample size to reach a definitive conclusion.

Our study included some limitations that are worth mentioning. First, CSF progranulin might not be a complete indicator of complex microglia activity which makes it hard to bring a definitive conclusion on the role of microglia in the AD pathophysiology. Second, more results are needed besides the ADNI database to provide more reliable data about participants with longer follow-up and variable imaging methods. In addition, this study considered both T and N as a single group which can decrease the heterogeneity in our study. Finally, the length of evaluation participants should be increased in further longitudinal studies to report more reliable results.

This study was the first attempt to evaluate the longitudinal association between CSF-progranulin and AD-imaging biomarkers including Aβ and tau in patients with early AD. Based on our findings there was no cross-sectional and longitudinal association between CSF progranulin and AD pathologic hallmarks in the course of the disease. In addition, the absence of significant difference in baseline level and change of CSF progranulin between A/T/N groups showed that there might be no distinctive roles for progranulin in AD-related pathologic changes. However, our findings revealed that an increase in CSF-progranulin over time is associated with faster formation of Aβ plaques in patients with only tau pathology based on the A/T/N classification (SNAP). Together, our findings showed that the role of progranulin-related microglial activity on AD pathology can be stage-dependent, complicated, and more prominent in non-AD pathologic changes. Thus, there is a need for further studies to consider progranulin-based therapies for AD treatment.

### Supplementary Information

Below is the link to the electronic supplementary material.

Supplementary file1 (DOCX 14 KB)

## Data Availability

The datasets analyzed during the current study are available upon request with no restriction. Please contact Dr. Fardin Nabizadeh (fardinnabizade1378@gmail.com) to access data.
