# Supplementary material for: Progranulin and neuropathological features of Alzheimer’s disease: longitudinal study
Source: Aging Clin Exp Res. 2024 Mar 5;36(1):55. doi: 10.1007/s40520-024-02715-9 (PMC10914850; doi:10.1007/s40520-024-02715-9)
Supplement: Supplementary file 1 — Supplementary file1 (DOCX 14 KB) [file 40520_2024_2715_MOESM1_ESM.docx]

| Supplementary 1. Number of participants with available CSF Progranulin and brain PET at each time points | | | | |
| --- | --- | --- | --- | --- |
| Variable | A-/TN- (n=246) | A+/TN- (n=166) | A+/TN+ (n=407) | A-/TN+ (n=182) |
| CSF Progranulin at baseline | 246 | 166 | 407 | 182 |
| CSF Progranulin at 12 months | 34 | 29 | 55 | 32 |
| CSF Progranulin at 24 months | 85 | 61 | 128 | 74 |
| CSF Progranulin at 36 months | 22 | 21 | 36 | 20 |
| CSF Progranulin at 48 months | 52 | 44 | 85 | 54 |
| Aβ-PET at baseline | 134 | 111 | 278 | 116 |
| Aβ-PET at 24 months | 91 | 78 | 199 | 71 |
| Aβ-PET at 48 months | 45 | 34 | 141 | 63 |
| tau-PET at baseline | 178 | 98 | 173 | 97 |
| tau-PET at 24 months | 76 | 55 | 108 | 43 |
| tau-PET at 48 months | 44 | 32 | 86 | 33 |
| Abbreviations: Aβ, Amyloid Beta; A, Aβ pathology; TN, Tau neurodegeneration; CSF, cerebrospinal fluid | | | | |
